# Methylmercury (MeHg) transcriptionally regulates NAD(P)H:quinone oxidoreductase 1 (NQO1) in Hepa-1c1c7 cells

**DOI:** 10.1016/j.crtox.2023.100126

**Published:** 2023-09-17

**Authors:** Mohammed A. Alqahtani, Mahmoud A. El-Ghiaty, Sara R. El-Mahrouk, Ayman O.S. El-Kadi

**Affiliations:** Faculty of Pharmacy and Pharmaceutical Sciences, University of Alberta, Edmonton, Alberta, Canada

**Keywords:** Methylmercury, Mercury, NQO1, NRF2, AHR, ARE

## Abstract

The detoxification of quinones through NAD(P)H:quinone oxidoreductase (NQO1) is a crucial mechanism to maintain cellular homeostasis. The exposure to heavy metals, specifically methylmercury (MeHg), induces several antioxidant enzymes, including NQO1. The nuclear factor erythroid 2-related factor-2 (NRF2) is known to regulate the expression of *Nqo1* gene and also the aryl hydrocarbon receptor (AHR) is another *Nqo1* gene regulator. This co-regulation prompted us to investigate which transcription factor (NRF2 or AHR) orchestrates the regulation of NQO1 expression upon MeHg exposure. Therefore, we investigated how MeHg can modulate the level of NQO1 expression by exposing Hepa-1c1c7 cells to several concentrations of MeHg with and without the addition of NQO1 inducers, DL-sulforaphane (SUL) and 2,3,7,8-tetrachlorodibenzo-p-dioxin (TCDD). We found that the mRNA expression *of Nqo1* is up-regulated by MeHg in time- as well as dose-dependent fashions. Additionally, MeHg increased the NQO1 at all expression levels with and without the presence of its inducers, SUL or TCDD. Furthermore, the MeHg-mediated increase of NQO1 expression was in parallel with a concurrent increase in the nuclear localization of NRF2 protein, but not that of AHR. Mechanistically, the antioxidant response element-driven reporter gene activity was induced by 215% upon MeHg exposure. Also, transfecting Hepa-1c1c7 with *Nrf2* siRNA reduced the MeHg-induced NQO1 protein expression by 60%. In conclusion, our findings provide evidence supporting the hypothesis that MeHg upregulates the *Nqo1* gene through a transcriptional mechanism at least in part via a NRF2-dependent mechanism.

## Highlights

• MeHg induces oxidative stress and that may affect NQO1 expression.
• MeHg enhances NRF2 nuclear accumulation and ARE transcriptional activation.
• NQO1 induction by MeHg is, at least in part, via NRF2 signaling pathway.

## Graphical abstract

**Figure:**
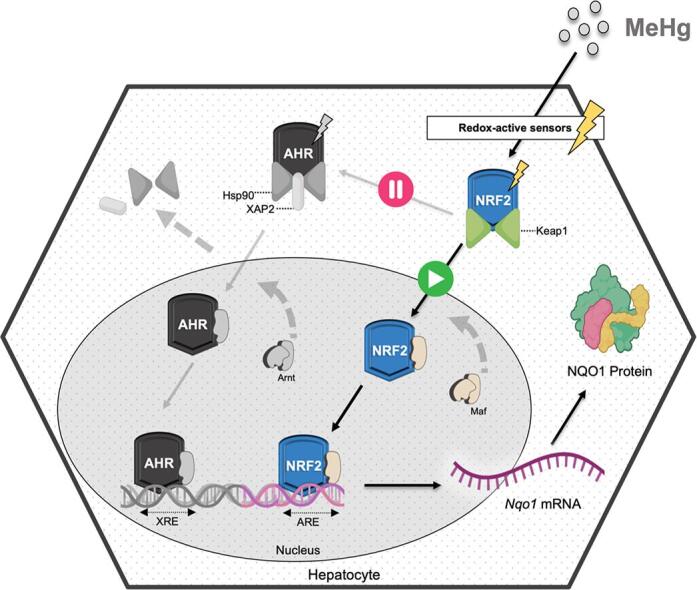

## 1 Introduction

Methylmercury (MeHg) is a reactive environmental pollutant that is mainly found in aquatic environments (Shaw and Chattopadhyay, 2020). It is produced through a process called biomethylation, which converts inorganic mercury (Hg) into MeHg (Compeau and Bartha, 1985). This process is primarily carried out by microorganisms in water, particularly from certain bacteria exposed to Hg released mostly from anthropogenic sources (Chételat et al., 2020, Raposo et al., 2020). MeHg has a remarkable ability to biomagnify in food chains, thus posing a serious health risk to human populations who consume fish and shellfish, which can bioaccumulate high levels of MeHg in their bodies (Romero-Romero et al., 2022). Due to its toxic properties, an array of studies has linked MeHg with environmental and health concerns (Chételat et al., 2020). A crucial facet of MeHg toxicity involves its interaction with cellular defense mechanisms, including the NQO1 enzyme (NAD(P)H dehydrogenase quinone 1), which plays a vital role in protecting cells from oxidative stress (Amara and El-Kadi, 2010).

The detoxification pathways of MeHg encompass a complex interplay of mechanisms within the liver and other tissues, with glutathione (GSH) conjugation being the most common. GSH directly binds to MeHg, neutralizing its harmful effects and facilitating the conversion of MeHg into less toxic metabolites, facilitating its subsequent elimination from the body (Ohsawa and Magos, 1974, Richardson and Murphy, 1975). On the other hand, enhancing NRF2-regulated antioxidant enzymes, including NQO1, plays a critical role in mitigating MeHg-induced oxidative stress, protecting cells and tissues from damage (Toyama et al., 2007). Moreover, NQO1 participates in the transformation of certain quinones into their antioxidant forms, contributing to cellular redox balance (Gong et al., 2008, Ross and Siegel, 2021).

The NQO1 enzymatic cycle, which occurs in the cytosol, involves a two-step process (ping-pong mechanism) where it initially accepts electrons from a substrate, typically a quinone compound, forming an enzyme-substrate complex. NQO1 then transfers these electrons to a cofactor, usually NAD(P)H, which regenerates the active form of NQO1. This redox cycling mechanism allows NQO1 to efficiently detoxify and stabilize various substrates, including important molecules like quinones and certain protein targets, contributing to its diverse cellular functions. The complexity associated with the NQO1 molecular roles has grown due to evidence indicating that NQO1 plays a role in preserving certain proteins from degradation (Adamovich et al., 2013, Oh et al., 2016). Many of these proteins have downstream regulation on their functionality or transcription, which suggests that NQO1 impact on a certain protein could lead to a magnified effect. A good example of this is how NQO1 stabilizes the tumor suppressor gene p53, preserving it from being degraded by 20S proteasome (Asher et al., 2002).

The expression of the NQO1 gene is controlled by the nuclear factor erythroid 2-related factor 2 (NRF2), which acts as a key regulator of myriad cellular signaling pathways and genes intricate in antioxidant and detoxification functions (Kim et al., 2021, Mills et al., 2018, Sajadimajd and Khazaei, 2018). The aryl hydrocarbon receptor (AHR) is another regulator that plays pivotal roles in various biological processes, including xenobiotic metabolism (Neavin et al., 2018, Trikha and Lee, 2020). NQO1 expression is regulated by a specific DNA sequence in its promoter region (motif), the antioxidant response element (ARE). Also, it has been reported that it is regulated by another motif, namely the xenobiotic response element (XRE). Accordingly, the ARE is recognized by NRF2, which binds to its sequence and upregulates NQO1 transcription in response to oxidative stress. On the other hand, the XRE is recognized by the aryl hydrocarbon receptor (AHR), which binds to its sequence and also upregulates NQO1 expression in response to xenobiotic exposure (Shaw and Chattopadhyay, 2020).The induction of NQO1 by an ARE inducer was found to be AHR-dependant, suggesting a potential interaction between the signaling pathways of ARE and XRE (Miao et al., 2005, Shaw and Chattopadhyay, 2020). Additionally, AHR ligands have been found to increase *Nrf2* mRNA transcript levels in mice, implying that AHR controls *Nrf2* (Miao et al., 2005). Conversely, NRF2 was reported to modulate AHR-regulated genes expression in Hepa-1c1c7 cells (Shin et al., 2007). Also, in Nrf2-deficient mice liver, a reduction in *AhR* mRNA levels was observed in comparison to the wild-type (Anwar-Mohamed et al., 2011, Shin et al., 2007). Therefore, these data suggest that NRF2 has a regulatory effect on AHR and its downstream targets. Furthermore, it was observed that *Keap1*-deficient mice exhibited an elevation in *AhR* mRNA levels, indicating a direct regulation of *AhR* by NRF2 (Shin et al., 2007).

Mercury and other heavy metals have been linked with NRF2/ARE activation which is a crucial defense mechanism for cellular protection against oxidative stress and toxic xenobiotics. Also, they have been found to be capable of modulating the induced AHR-regulated genes expression in various cell lines (Alqahtani et al., 2023, El-Ghiaty et al., 2022, El-Ghiaty et al., 2023, Xu et al., 2019, Zhou et al., 2019). Previously, we reported that Hg induced NQO1 expression *in vivo* (Amara et al., 2012), and that finding was further demonstrated to be possibly through NRF2/ARE pathway *in vitro* (Amara and El-Kadi, 2010). Therefore, investigating the NRF2/AHR regulatory mechanism in the expression of NQO1 in response to MeHg exposure is an important area of research, as it can provide insights into the mechanism of MeHg-mediated toxicity, thus enabling the development of new strategies for protecting against the toxic metal exposure. Our hypothesis is that MeHg can induce the Nqo1 gene expression by activating the signaling pathways of NRF2 and AHR, either independently or collectively. Therefore, the primary goal of this study was to examine the MeHg-mediated transcriptional regulation on NQO1 expression.

## 2 Materials and methods

### 2.1 Materials

MEM α (Minimum Essential Medium α), protease inhibitor cocktail, Methylmercury(II) chloride, DL-Sulforaphane and 3-(4,5-dimethylthiazol-2-yl)-2,5-diphenyltetrazolium bromide (MTT) are from Sigma Chemical Co. (St. Louis, MO). ARE Reporter Kit (60514) is from BPS Bioscience (San Diego, CA, USA). Nuclear and Cytoplasmic Extraction Reagents (NE-PER™) is from Thermo Fisher Scientific (Waltham, MA). Anti-mouse IgG HRP-linked 2ry antibody (7076) is from Cell Signaling Technology (Danvers, MA, USA). NQO1 activity kit (ab184867) is Abcam (Cambridge, UK). Mouse monoclonal anti-NQO1 (sc-32793), anti-NRF2 (sc-365949), anti-LAMIN B1 (sc-377000), *Nrf2* siRNA (sc-37049), siRNA transfection medium (sc-36868), siRNA transfection reagent (sc-29528) control siRNA-A (sc-37007) are from Santa Cruz Biotechnology (Santa Cruz, CA). All remaining materials are from Fisher Scientific (Waltham, MA, USA). Further details about the used materials not mentioned above can be found in our previously published paper (Alqahtani et al., 2023).

### 2.2 Biohazard precaution

Since methylmercury and 2,3,7,8-tetrachlorodibenzo-p-dioxin (TCDD) are both highly toxic substances, the recommendations of the University of Alberta's Office of Environmental Health and Safety were followed by ensuring that all personnel received secure handling procedures training, used appropriate personal protective equipment (PPE), and disposed of contaminated items properly.

### 2.3 Tissue culture and chemical treatment

Murine hepatoma cells (Hepa-1c1c7) were obtained from American Type Culture Collection (ATCC® CRL-2026), they were cultured in MEMα with the addition of fetal bovine serum (10% FBS) on a T-75 cell culture flask inside an incubator that set up for 5% CO2 level at 37 °C. MeHg with following concentrations: 1.25, 2.5, and 5 μM with and without the presence of either TCDD (1 nM) or sulforaphane (5 μM) were used to treat the cells. A freshly prepared MeHg solution was dissolved in Milli-Q water, while both SUL and TCDD were prepared in dimethyl sulfoxide (DMSO). In all experiments, the utilized DMSO concentrations were no more than 0.01% (v/v). The results of the cell viability assessment were used to select the feasible concentration of MeHg and SUL.

### 2.4 Extraction of RNA and synthesis of single-stranded cDNA

TRIzol reagent was utilized to extract the Hepa-1c1c7 cells total RNA. The obtained RNA quantity and quality were measuring by a microplate reader (Biotek Synergy H1). Afterward, the single-strand cDNA synthesis was created by using cDNA reverse transcription kit.

### 2.5 Quantification and data analysis of real-time PCR

To quantify a specific expression of a tested mRNA, real-time PCR was employed by amplifying the resulting single-stranded cDNA using the QuantStudio 5. More details about the real-time reaction and data calculation are described in our previous paper (Alqahtani et al., 2023). For primers sequence see (Table 1).

**Table 1:** Mouse primers sequence.

Gene	Forward primer (F)	Reverse primer (R)
*β-actin*	5′TAT-TGG-CAA-CGA-GCG-GTT-CC3′	5′*GGC-ATA-GAG-GTC-TTT-ACG-GAT-GTC*3′
*Ahr*	5′CGG-CTT-CTT-GCA-AAA-CAC-AGT-3′	5′GTA-AAT-GCT-CTC-GTC-CTT-CTT-CAT-C3′
*Nrf2*	5′CGA-GAT-ATA-CGC-AGG-AGA-GGT-AAG-A3′	5′GCT-CGA-CAA-TGT-TCT-CCA-GCT-T3′
*Nqo1*	5′*GCA-GGA-TTT-GCC-TAC-ACA-ATA-TGC*3′	5′AGT-GGT-GAT-AGA-AAG-CAA-GGT-CTT-C3′

### 2.6 Western blot

A previously described method was used to carry out the western blot analysis (Alqahtani et al., 2023). Briefly, 100 μg of total cell lysates were electrophoresed on 10% SDS-PAGE gel, followed by transfer onto a PVDF membranes, then incubate them at 4 °C for at least 1 h in a blocking solution comprised 0.5% Tween-20, 3 mM potassium chloride, 0.15 M sodium chloride, 2% bovine serum albumin (BSA), 25 mM Tris-base (TBS), and skim milk (5%). Afterward, the membranes were incubated with primary antibodies for at least 2 h at 4 °C. Mouse monoclonal anti-GAPDH (1:5000), anti-NQO1 (1:500), anti-NRF2 (1:500), and anti-LAMIN B1 (1:100) were used. Then, blots were incubated for 1 h with the secondary antibody, anti-mouse IgG HRP-linked secondary antibody (1:2000). The enhanced chemiluminescence technique was employed to visualize the bands, following the manufacturer's instructions. The ChemiDoc MP imaging system (Bio-Rad Laboratories, Hercules, CA, USA) were utilized to quantify the protein band intensity.

### 2.7 Catalytic activity of NQO1 enzyme

NQO1 activity assay kit was used to measure the NQO1 enzymatic activity. 20 μg of cellular lysate protein was used for the reaction. After that the fluorescence was measured by 20 sec intervals at 440 nm absorbance in Synergy H1 plate reader. The replicates were combined to calculate the average, and NQO1 activity was expressed as the mOD 440 nm without dicoumarol following subtraction of mOD 440 nm with dicoumarol.

### 2.8 Determination of NQO1 protein stability

To assess the NQO1 protein stability, the cycloheximide-chase (CHX-chase) experiment was conducted to determine the MeHg-mediated effect of on NQO1 protein half-life. Before exposing cells to MeHg, they were first pretreated for 24 h with TCDD (1 nM). After adding CHX (10 μg/mL) to halt protein biosynthesis, cells were exposed to MeHg (5 μM). Total protein from cell lysates were obtained at 0, 6, 12, and 24 h after exposure to MeHg. The amount of proteins were determined using the Lowry method, while the protein of NQO1 was detected by western blot.

### 2.9 Determination of ARE-luciferase reporter activity

ARE reporter kit was used to measure the ARE-luciferase activity (BPS Bioscience). It comprises a transfection-ready reporter vectors for ARE luciferase. This reporter features a firefly luciferase gene and Renilla luciferase vector (used as an internal control). Also, a non-inducible firefly luciferase vector was mixed with Renilla luciferase vector, to serve as the reaction negative control.

### 2.10 Nuclear protein extract preparation

MeHg was incubated at 0, 1, 2, 3, 4, 5, 6, and 24 hr with Hepa-1c1c7 cells. After that, NE-PER^TM^ kit was used according to the provided instructions (Thermo Fisher Scientific) to obtain nuclear protein fraction.

### 2.11 Nrf2 siRNA transfection

Hepa-1c1c7 cells were transfected for 24 h with either the negative control or Nrf2 siRNAs according to provided instructions by Santa Cruz Biotechnology. To assess the efficiency of transfection, the levels of NRF2 mRNA and protein expression were measured, cells were eposed to MeHg (5 µM) for 24 h and then assayed for Real-time PCR or Western blot.

### 2.12 Statistical analysis

The data are presented as the mean ± SEM (standard error of the mean). A comparative analysis was conducted between the results obtained from all experimental groups and their respective control groups using GraphPad Prism software (GraphPad Software Inc., San Diego, CA). The independent *t*-test was performed to determine the statistical difference between two groups. Additionally, the one-way analysis of variance (ANOVA) followed by the Dunnett test was conducted to determine the statistical difference between more than two groups. The differences were considered significant when *p* < 0.05.

## 3 Results

### 3.1 Determination of Hepa-1c1c7 cell viability

**Fig. 1:**
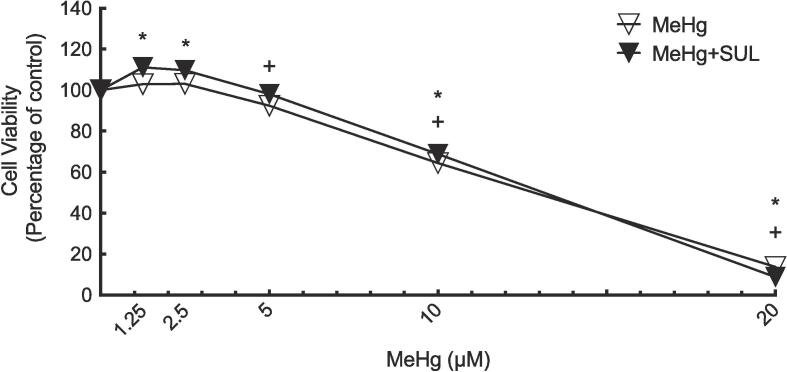
Co-exposure effect of MeHg and SUL on Hepa-1c1c7 cell viability.

The feasible concentrations of MeHg in this study was determined by exposing Hepa-1c1c7 cells to several concentrations of MeHg (1.25, 2.5, 5, 10, and 20 µM) with or without the presence of 5 µM SUL for 24 h period, then the MTT assay was used to evaluate the viability of cells (Fig. 1). Data showed that 1.25–5 µM of MeHg concentrations did not remarkably reduce the viability Hepa-1c1c7 cells, either with or without 5 µM SUL. Therefore, these concentrations were selected for use in all subsequent experiments.

Increasing concentrations of MeHg (1.25, 2.5, 5, 10 and 20 μM) were incubated with Hepa-1c1c7 cells. Values in Fig. 1 are shown as the percentage from control cell viability. The symbols (+) or (*) denote statistical significance between the “MeHg group” or “MeHg + SUL group” and the control group.

### 3.2 Determination of *Nqo1* mRNA levels upon MeHg exposure

**Fig. 2:**
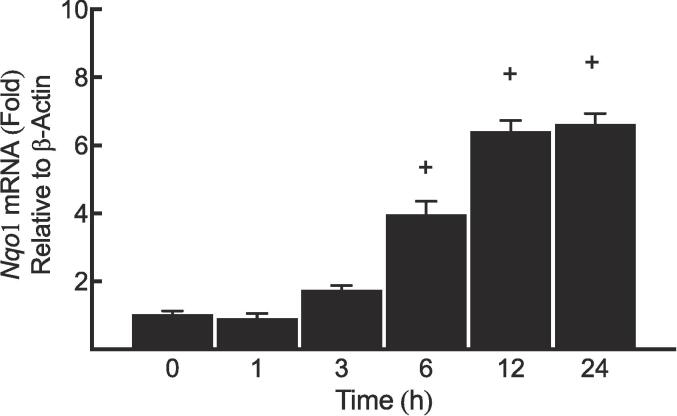
Determination of *Nqo1* mRNA levels upon MeHg exposure.

To investigate how the *Nqo1* mRNA levels change over time upon MeHg exposure, MeHg (5 µM) was used to treat Hepa-1c1c7 at 0, 1, 3, 6, 12, and 24 h. As shown in Fig. 2, MeHg caused a gradual increase in the mRNA transcripts of *Nqo1*. Specifically, after 6, 12, and 24 hr of MeHg exposure, the *Nqo1* mRNA levels were induced, with respective increments of 3-, 5-, and 5.1-fold compared to the 0 h group.

Several time points (0, 1, 3, 6, 12 and 24 h) were used to incubate MeHg (5 μM) with Hepa-1c1c cells. Results are expressed as a fold of induction. The symbol (+) indicates significant difference of a time point compared to “0h group”.

### 3.3 Determination of the basal and inducible (TCDD-mediated) NQO1 levels upon MeHg exposure

**Fig. 3:**
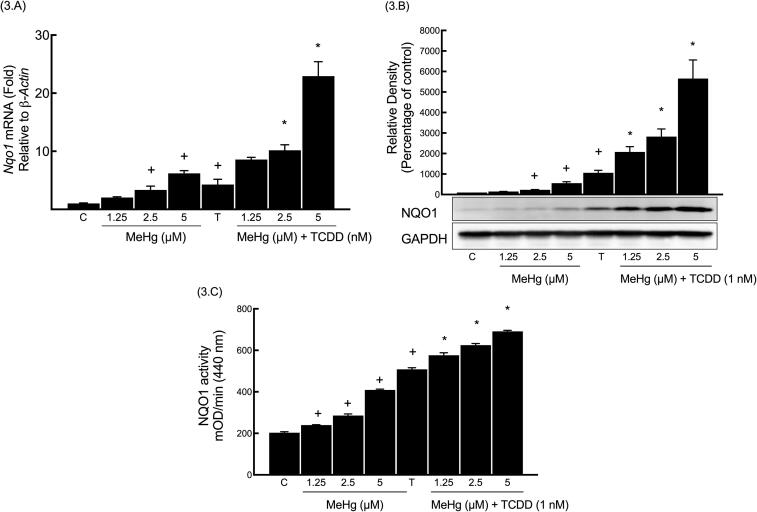
Determination of the basal and inducible (TCDD-mediated) NQO1 levels upon MeHg exposure.

The basal and inducible (TCDD-mediated) levels of *Nqo1* mRNA were examined by exposing Hepa-1c1c7 cells to varying MeHg concentrations with and without TCDD (1 nM). Results showed that MeHg at 2.5 and 5 μM increased the *Nqo1* mRNA expression, with respective increments of 229% and 507% compared to the control group. Furthermore, the *Nqo1* mRNA level was significantly increased by 319% when cells were treated with TCDD alone. The addition of 2.5 μM and 5 μM MeHg to TCDD treatment further induced the *Nqo1* level, with respective increments of 137% and 435% compared to the TCDD group, as shown in Fig. 3.A.

Hepa-1c1c7 cells were exposed to similar MeHg concentrations, but for 24 h period. Here we attempted to examine if the MeHg-mediated increase in *Nqo1* mRNA leads to changes in the catalytic activity and protein. Similar to the *Nqo1* mRNA findings, MeHg was able to gradually induce the basal NQO1 protein expression (Fig. 3.B), with statistically significant changes observed only at 2.5 μM and 5 μM, with respective increments of 129% and 455% compared to the control group. Also, NQO1 catalytic activity (Fig. 3.C) was also increased at 1.25 μM, 2.5 μM, and 5 μM, with respective increments of 17%, 40%, and 101% compared to the control group. Furthermore, TCDD treatment significantly enhanced the NQO1 levels of protein and activity, with respective increments of 959% and 150% compared to control group. Moreover, all the tested MeHg concentrations significantly enhanced the TCDD-induced NQO1 protein expression. The enhancement was significant at 1.25, 2.5, and 5 μM, with respective increments of 95%, 166%, and 434% for protein expression and 13%, 22%, and 35% for enzymatic activity compared to TCDD group.

Several MeHg concentrations (1.25, 2.5, and 5 μM) were used to treat Hepa1c1c-cells with or without 1 nM TCDD. Real-time PCR (A), western blot (B) and NQO1 activity assay (C) were carried out as outlined in methods section. The symbol (+) denotes statistical significance between the “MeHg group” and the control group, while the symbol (*) denotes statistical significance between the “MeHg + TCDD group” and the TCDD group.

### 3.4 Determination of the basal and inducible (SUL-mediated) NQO1 levels upon MeHg exposure

**Fig. 4:**
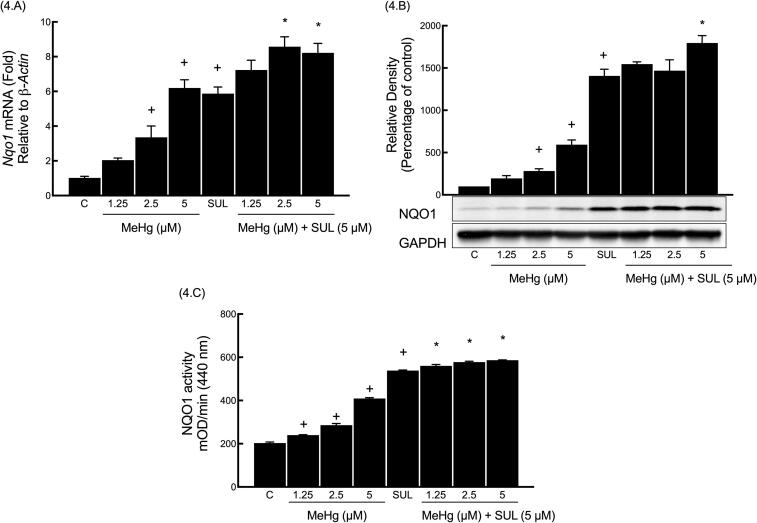
Determination of the basal and inducible (SUL-mediated) NQO1 levels upon MeHg exposure.

The basal and inducible (SUL-mediated) levels of *Nqo1* mRNA were examined by exposing Hepa-1c1c7 cells to varying MeHg concentrations with and without SUL (5 μM). Results showed that MeHg at 2.5 and 5 μM induced the *Nqo1* mRNA expression, with respective increments of 229% and 507% compared to the control group. Furthermore, the *Nqo1* mRNA level was significantly increased by 475% when cells were treated with SUL alone. The addition of 2.5 μM and 5 μM MeHg to SUL treatment further induced the *Nqo1* level, with respective increments of 45% and 40% compared to the SUL group, as shown in Fig. 4.A.

Hepa-1c1c7 cells were exposed to similar MeHg concentrations, but for 24 h period. Here we attempted to examine if the MeHg-mediated increase in *Nqo1* mRNA leads to changes in the catalytic activity and protein. Similar to the *Nqo1* mRNA findings, MeHg was able to gradually induce the basal NQO1 protein expression (Fig. 4.B), with statistically significant changes observed only at 2.5 μM and 5 μM, with respective increments of 181% and 493% compared to the control group. Moreover, NQO1 catalytic activity (Fig. 4.C) was also increased in a at 1.25 μM, 2.5 μM, and 5 μM, with respective increments of 17%, 40%, and 101% compared to the control group. Furthermore, SUL significantly enhanced the NQO1 protein and activity levels, with respective increments of 1307% and 165% compared to control group. Moreover, MeHg at the highest concentration (5 μM), significantly enhanced the SUL-induced NQO1 protein expression by 27% compared to SUL group. However, MeHg at the concentrations 1.25, 2.5 and 5 μM slightly potentiated the SUL-induced NQO1 activity, with respective increments of 4%, 7% and 9% compared to the SUL group.

Several MeHg concentrations (1.25, 2.5, and 5 μM) were used to treat Hepa1c1c-cells with or without the presence of 5 μM SUL. Real-time PCR (A), western blot (B) and NQO1 activity assay (C) were carried out as as outlined in methods section. Results are expressed as a fold of induction, The symbol (+) indicates statistical significance between the “MeHg group” and the control group, while the symbol (*) indicates a statistical significance between the “MeHg + SUL group” and the SUL group.

### 3.5 Determination of the NQO1 protein stability upon MeHg exposure

**Fig. 5:**
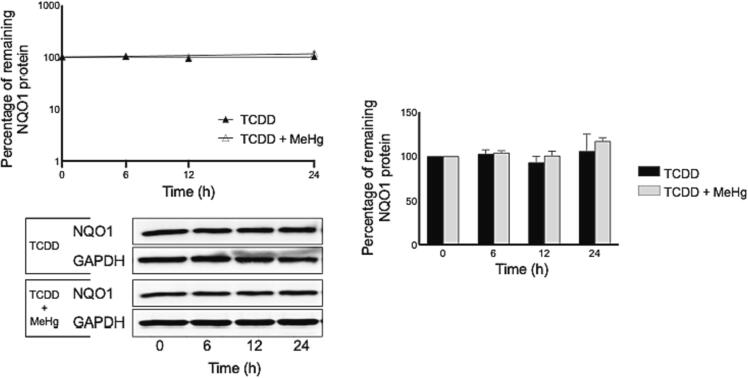
Determination of the NQO1 protein stability upon MeHg exposure.

The marked increase in the level of *Nqo1* mRNA (as shown in Fig. 5), led to additional investigation to examine if MeHg can alter the stability of NQO1 protein. Therefore, we preformed the cycloheximide chase to evaluate the MeHg impact on the protein half-life of NQO1. Results revealed that MeHg did not significantly change the NQO1 protein expression over the 24 h period, thus indicating that NOQ1 protein half-life is more than 24 h (Fig. 5). Taken together, these findings suggest that MeHg do not alter NQO1 protein stability through a post-translational mechanism.

Results are expressed as percentage from 0 h group (100%), mean ± SEM (n = 6). CHX chase experiment was carried out as described under materials and methods section. The symbols (+) or (*) indicate statistical significance between the “MeHg group” or “MeHg + TCDD group” and the “0h group”.

### 3.6 Effect of MeHg on *Nqo1* gene transcription

**Fig. 6:**
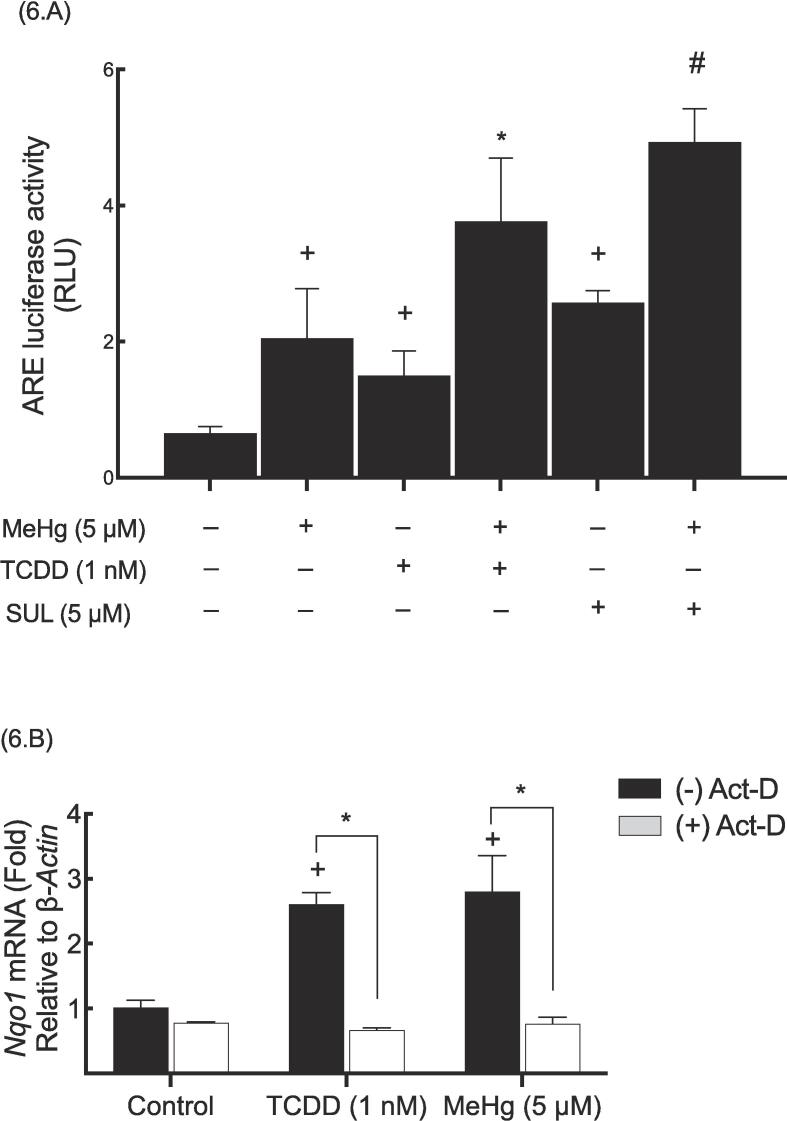
Effect of MeHg on Nqo1 gene transcription.

Transfecting Hepa-1c1c7 cells with ARE-driven reporter gene was employed to examine the involvement of the ARE activation in the induced NQO1 expression by MeHg. Data indicated that MeHg exposure (5 μM) increased the activity of ARE-driven luciferase, with respective increment of 215% compared to the control group (as depicted in Fig. 6A). Additionally, 1 nM TCDD and 5 μΜ SUL led to a substantial increase in activity levels, with respective increments of 130% and 296% compared to the control group. However, when MeHg (5 μΜ) was combined with either TCDD or SUL treatment, ARE-luciferase activity was increased, with respective increments of 83% and 72% compared to group treated with SUL of TCDD groups.

A transcription inhibitor, actinomycin D (Act-D), that blocks the *de novo* synthesis of *Nqo1* was employed to assess the involvement of a transcriptional regulation mechanism. Dara showed that the basal expression of *Nqo1* mRNA was unaltered with the treatment of Act-D (Fig. 6.B). Conversely, MeHg and TCDD increased *Nqo1* mRNA levels, with respective increments of 72% and 78%. However, the MeHg- and TCDD-mediated inductions were completely abolished when cells treated with Act-D.

MeHg (5 μM) with and without either TCDD (1 nM) or SUL (5 μM) were used to treat Hepa1c1c-cells. ARE-luciferase activity (A) and real-time PCR (B) were carried out as outlined in methods section and data are expressed as a fold of induction. The symbol (+) indicates a statistical significance between the “MeHg-, TCDD- or SUL- treated groups”“ and the control group, while the symbol (*) or (#) indicate statistical significance between the ”MeHg + TCDD group“ and the “TCDD group” or “MeHg + SUL group” and the “SUL group”, respectively.

### 3.7 Determination of the time-dependent changes in *Nrf2* and *AHR* mRNA levels upon MeHg exposure

**Fig. 7:**
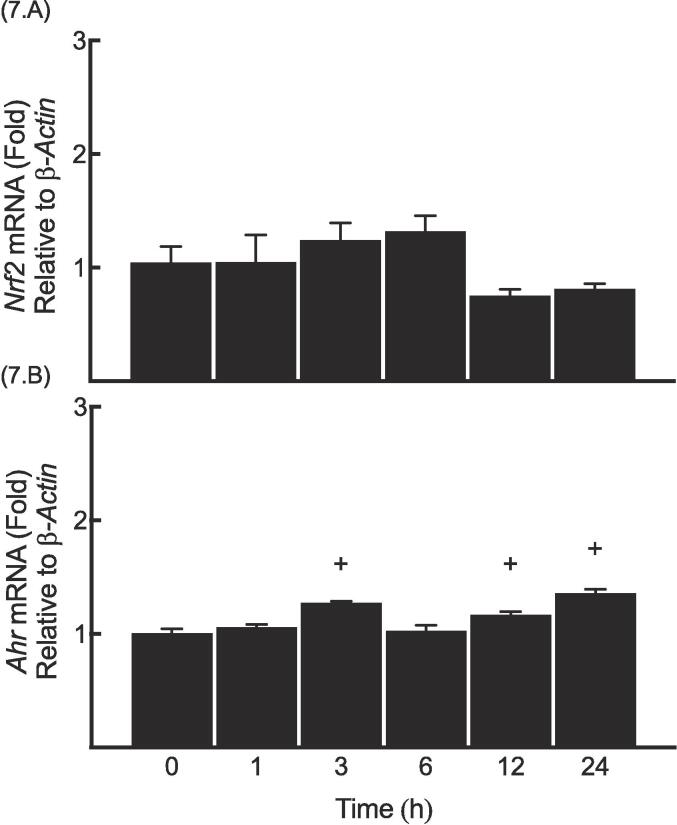
Determination of the time-dependent changes in *Nrf2* and *Ahr* mRNA levels upon MeHg exposure.

As a result of the observed increase in basal NQO1 expression by MeHg, we proceeded to investigate the impact of MeHg on the mRNA levels of *Nrf2* and *Ahr*. Therefore, MeHg (5 µM) was used to treat Hepa-1c1c7 cells at 0, 1, 3, 6, 12, and 24 h. Based on the findings obtained from this experiment, MeHg was unable to alter the level of *Nrf2* mRNA (Fig. 7.A), while it caused a modest increase in the *Ahr* mRNA transcripts at 3, 12 and 24 h compared to 0 h group (Fig. 7.B).

MeHg (5 μM) was used to treat Hepa1c1c-cells for the time points indicated. Real-time PCR experiments for *Nrf2* (A) and *Ahr* (B) were carried out as outlined in methods section and results are expressed as a fold of induction. The symbol (+) indicates significant difference of a time point compared to “0h group”.

### 3.8 Determination of the nuclear NRF2 and AHR proteins upon MeHg exposure

**Fig. 8:**
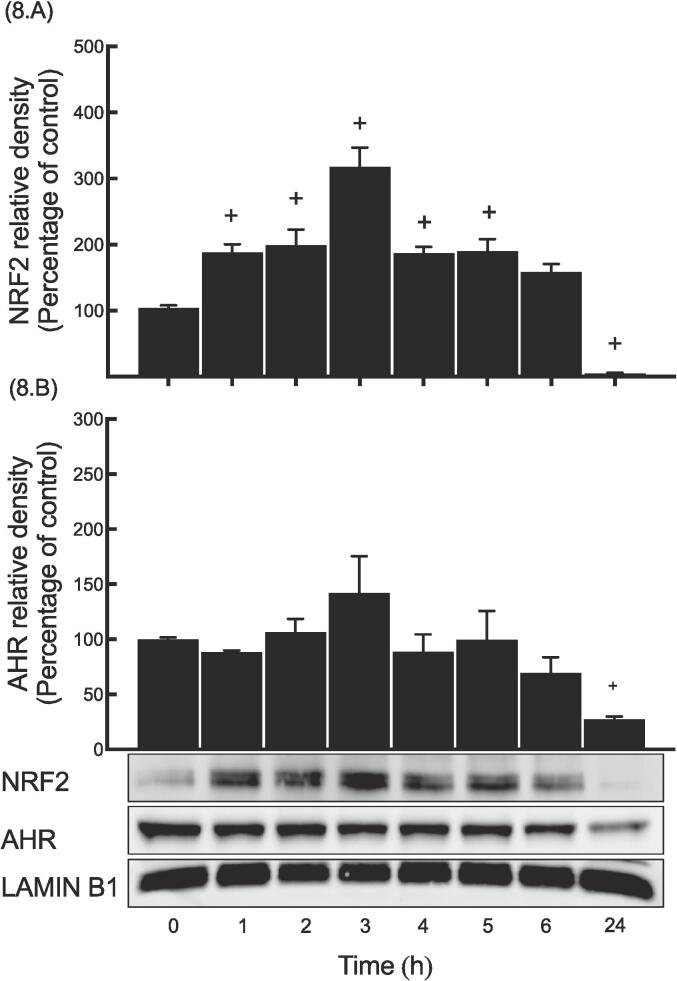
Determination of the nuclear NRF2 and AHR proteins upon MeHg exposure.

At different time points, MeHg 5 µM was used to the cells to assess the nuclear levels of NRF2 and AHR. Data showed that MeHg significantly increased the NRF2 nuclear accumulation at 1 and 2 h, with respective increments of 84% and 95% (Fig. 8.A). However, the maximum NRF2 nuclear accumulation level was observed at 3 h by 213% followed by a gradual decline in the nuclear shuttling at 4, 5 and 6 h, by 83%, 86% and 29%, respectively, while it was completely abolished at 24 h. In contrast, MeHg did not alter the AHR nuclear accumulation at time-points tested from 1 to 6 h, while it significantly reduced its expression below the basal level at 24 h by 72% (Fig. 8.B).

MeHg (5 μM) was used to treat Hepa1c1c-cells for the time points indicated. Western blot experiments were carried out as outlined under materials and methods section. NRF2 (A) and AHR (B) nuclear proteins are expressed as a fold of induction. The symbol (+) indicates significant difference of a time point compared to “0h group”.

### 3.9 Nrf2 siRNA effect on NQO1 expression levels

**Fig. 9:**
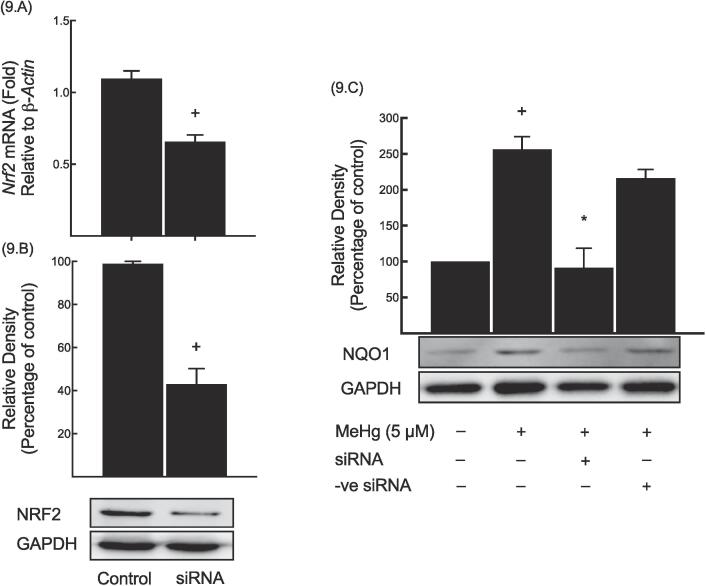
*Nrf2* siRNA effect on NQO1 expression levels.

The increased nuclear NRF2 accumulation prompted us to employ siRNA targeting mouse *Nrf2* and evaluated the transfection efficiency by analyzing NRF2 expression. Results indicated that Nrf2 siRNA caused a marked decrease in both Nrf2 mRNA (Fig. 9.A) and protein expression (Fig. 9.B) levels by 40% and 56%, respectively, compared to control. Conversely, NQO1 expression was significantly increased by MeHg, by 156% when compared to control (Fig. 9.C). Interestingly, siRNA *Nrf2* significantly reduced the MeHg-induced NQO1 expression by 64% compared to MeHg group. However, transfecting the cells with the siRNA negative control was unable to significantly alter the MeHg-induced NQO1 expression when compared to MeHg group.

A specific siRNA targeting mouse *Nrf2* or a negative-control siRNA were used to transfect Hepa-1c1c7 cells, followed by exposure to 5 μM of MeHg. *Nrf2* mRNA (A), NRF2 protein (B) and NQO1 protein (C) were carried out as outlined in methods section and data are expressed as a fold of induction. The symbols (+) and (*) denote to statistical significance when compared to the control and MeHg groups, respectively.

## 4 Discussion

Since the liver plays a crucial role in detoxification and metabolism, murine hepatoma Hepa-1c1c7 cells were employed in this study which makes them a suitable model for studying liver-specific processes. Additionally, their high inducibility for AHR-regulated enzymes, including NQO1, made them the ideal choice for investigating the complexed mechanisms underlying xenobiotic metabolism and detoxification responses (El-Sayed et al., 2007, Fahey et al., 2004).

Studies have shown that NQO1 activity is increased in response to mercury exposure, likely as a protective response to help eliminate the toxic metal-mediated effects (Ni et al., 20102010, Zhao et al., 2021). However, excessive exposure to mercury can overwhelm the detoxification system, leading to detrimental health outcomes (Kondo, 2000). However, we previously demonstrated that inorganic mercury (Hg) is capable to modulate the NQO1 basal and inducible levels via regulatory mechanisms (Amara and El-Kadi, 2011, Korashy and El-Kadi, 2006, Korashy and El-Kadi, 2008). Given that the NQO1 expression is co-regulated by two transcription factors, NRF2 and AHR, we hypothesized that the organic mercury (MeHg) up-regulates the expression of *Nqo1* gene by activating NRF2 and AHR signaling pathways, either independently or collectively. Therefore, we aimed to examine the MeHg-mediated effect on the basal as well as the inducible levels of NQO1 expression by TCDD, a bifunctional inducer, and SUL, a monofunctional inducer. Also, to mechanistically investigate which transcription factors, namely NRF2 and AHR, are involved in NQO1 regulatory pathway upon MeHg exposure.

In this study, MeHg exhibited a rapid up-regulation in the basal *Nqo1* mRNA, starting from 6 h of exposure. (Fig. 2). In addition, MeHg increased the inducible (SUL- and TCDD-mediated) NQO1 expression at all levels in murine Hepa-1c1c7 cell line.

These findings are consistent with previous report that has demonstrated that exposure to inorganic mercury can result in elevated levels of basal and inducible NQO1 mRNA, protein, and activity in human HepG2 cell line (Amara and El-Kadi, 2011). Therefore, it was of interest to compare our findings with those obtained from human cell line in order to identify any species-specific effects.

Since the NQO1 expression is co-regulated by NRF2 and AHR and their respective DNA motifs, ARE and XRE, we found several pieces of evidence suggesting a potential involvement of a transcriptional mechanism in the induced NQO1 expression by MeHg. First, MeHg up-regulated both the basal and the inducible (SUL- and TCDD-mediated) *Nqo1*. Second, the presence of Act-D, an RNA synthesis inhibitor, completely eliminated MeHg-mediated increase in *Nqo1* mRNA.

This suggests that MeHg enhances the *de novo* synthesis of *Nqo1* RNA, similar to the effect observed with TCDD, which induces this gene transcriptionally through XRE-ARE pathway. Third, MeHg was capable of increasing basal and inducible ARE-luciferase activity, however, we previously reported that MeHg did not alter XRE-luciferase activity using same cell line (Alqahtani et al., 2023). Therefore, these findings imply that ARE (or NRF2) pathway, but not that of XRE (or AHR), may be solely involved in the NQO1 transcriptional regulation upon MeHg exposure.

Although the increased levels of basal and inducible *Nqo1* mRNA in response to MeHg suggest that a transcriptional mechanism might be involved, it is also possible that a post-translational mechanism may play a role. To investigate this possibility, a CHX chase experiment was conducted to determine whether MeHg affects NQO1 protein stability. If MeHg increased the protein stability, one would expect the NQO1 protein half-life to increase. However, according to our findings the post-translational mechanisms is unlikely to play a role in the NQO1 protein regulation as MeHg did not significantly affect its half-life. Moreover, the data presented in this paper showed that the half-life of both the basal and induced NQO1 protein is longer than 24 h, which is consistent with previous reports (Korashy and El-Kadi, 2006, Siegel et al., 2001).

To determine if MeHg up-regulates *Nqo1* gene by inducing its transcriptional regulators (NRF2 and AHR), we evaluated the MeHg-mediated effect on the expression of *Nrf2* and *Ahr* mRNAs at several time points.

Data showed that MeHg failed to exhibit a notable effect on the mRNA level of Nrf2, however, a minor increase in *Ahr* was observed. In contrary to our results, MeHg, and other mercurial, were reported not to alter *Ahr* mRNA in Kunming mice and other cell lines, and that can be likely attributed to the cellular responses to oxidative stress as well as the feedback loop controlling of *Ahr* steady-state level (Kou et al., 2022, Morel et al., 1999, Xu et al., 2016).

Although MeHg treatment did not affect the transcription of Nrf2, it was still worth examining whether MeHg could enhance the nuclear translocation of NQO1 regulators, specifically NRF2 and AHR. Our findings revealed that as early as 1 h after treatment, MeHg significantly increased NRF2 nuclear accumulation that peaked at 3 h and subsequently declined gradually, while AHR levels remained unchanged until being degraded at 24 h.

This study shows that the activation of NRF2, but not that of AHR, is required for MeHg-induced activation of the *Nqo1* gene through the binding of NRF2 complex to its DNA motif (ARE) and subsequent transcriptional induction of *Nqo1*.

To gain further insight into the MeHg-mediated induction of NQO1 and the possible involvement of *Nrf2* in this regulatory mechanism, we used siRNA to silence *Nrf2*. The results showed that the basal and MeHg-induced *Nqo1* gene expression was reduced in the presence of *Nrf2* siRNA. (Fig. 9.C). The data provided suggest that the MeHg-mediated modulation of NQO1 expression is regulated by a mechanism that is dependent on NRF2.

## 5 Conclusion

The induction of NQO1 by MeHg is, at least in part, NRF2-mediated and this was supported by a series of observations, including the fact that MeHg induces the transcriptional activation of the ARE luciferase activity and enhances NRF2 nuclear accumulation. Also, knocking-down *Nrf2* RNA notably reduces the MeHg-mediated NQO1 expression. By examining the MeHg-mediated effect on the expression of NQO1, this study contributes to the overall understanding of how MeHg could impact cellular responses to oxidative stress.

## CRediT authorship contribution statement

**Mohammed A. Alqahtani:** Methodology, Formal analysis, Investigation, Writing – original draft, Writing – review & editing. **Mahmoud A. El-Ghiaty:** Investigation, Writing – original draft. **Sara R. El-Mahrouk:** Investigation, Writing – original draft. **Ayman O.S. El-Kadi:** Writing – review & editing, Supervision, Funding acquisition.

## Declaration of Competing Interest

The authors declare that they have no known competing financial interests or personal relationships that could have appeared to influence the work reported in this paper.

## Data Availability

Data will be made available on request.
